# Reporting of Salmonellosis Markedly Declined in Finland During 28 Years of Surveillance, 1995–2022

**DOI:** 10.3390/microorganisms13030693

**Published:** 2025-03-20

**Authors:** Kristiina Suominen, Jukka Ollgren, Elina Leinonen, Ruska Rimhanen-Finne

**Affiliations:** 1 Department of Public Health, Finnish Institute for Health and Welfare, Mannerheimintie 166, 00300 Helsinki, Finland; kristiina.suominen@thl.fi (K.S.); jukka.ollgren@thl.fi (J.O.); 2 Food Safety Department, Finnish Food Authority, Mustialankatu 3, 00790 Helsinki, Finland; elina.leinonen@ruokavirasto.fi

**Keywords:** *Salmonella* infections, gastroenteritis, travel, register, demography, foodborne outbreaks, Finland, humans

## Abstract

Salmonellosis is the second most common foodborne bacterial gastroenteritis in humans in Finland and worldwide. In Finland, a national salmonella control program covering cattle, pigs, and poultry, as well as the meat and eggs obtained from them, has been in place since the country joined the EU in 1995. To identify trends in the incidence of salmonellosis and to reflect on their causes, we analyzed *Salmonella* case data from the Finnish Infectious Disease Register (FIDR) from 1995 to 2022 and outbreak data from the national food and waterborne outbreak register (the FWO register) in the period 2010–2022. From 1995–1999 to 2015–2019, the incidence of salmonellosis decreased by 66% and 63% for domestic and travel-related cases, respectively. Most salmonellosis cases (72%) were travel-related, and, of them, 27% were infected in Southeast Asia. The most common serovars were *S*. Typhimurium and *S*. Enteritidis (38% and 19% of domestic cases, and 7% and 39% of travel-related cases). During 2010–2022, *Salmonella* sp. was reported as a cause in 31 foodborne outbreaks. In 14 of them, the source was identified at foodstuff level, and 12 sources were of foreign origin. The results of this study indicate that the national salmonella control program may have prevented domestic human infections in Finland.

## 1. Introduction

Salmonellosis is the second most common foodborne bacterial gastroenteritis in humans and a major cause of foodborne outbreaks in the EU/EEA area [1]. According to the World Health Organization, salmonellosis is one of the top four global causes of diarrhea [2].

Typical symptoms of salmonellosis include diarrhea, vomiting, and nausea [3]. Also, fever, abdominal pain, headache, and myalgia may be present. The incubation period is around 6–48 h, and the symptoms usually cease in 3–7 days without treatment [4]. However, bacteremia may sometimes develop and is predisposed by certain serotypes, immunosuppression, and the age of the patient [3]. *Salmonella* transmits by the fecal–oral route, and many animals can act as a reservoir for *Salmonella*. Humans most often contract the infection through contaminated food, contaminated water, or through contact with infected humans or animals [4].

When Finland joined the EU in 1995, a national salmonella control program was approved by the Commission due to the low prevalence of *Salmonella* in Finnish animals and animal-derived foods [5]. The control program covers cattle, pigs, and poultry, as well as the meat and eggs obtained from them [5,6]. In addition, Finland received special guarantees regarding *Salmonella*, which means that imported batches of meat and eggs from countries without a similar control program must be tested for *Salmonella* with negative results in the country of origin [7,8]. The prevalence of *Salmonella* in poultry, pigs, and cattle in Finland has remained below the target threshold of 1%, even though, in recent years, the case numbers in cattle and pig farms have increased [9].

To identify trends in the incidence of salmonellosis and reflect on their causes, we report demographics of *Salmonella* cases reported to the Finnish Infectious Disease Register (FIDR) during the period 1995–2022 and foodborne outbreaks caused by *Salmonella* sp. during the period 2010–2022.

## 2. Materials and Methods

### 2.1. Salmonella Surveillance

In Finland, salmonellosis is classified as a generally hazardous communicable disease, and laboratory-confirmed salmonellosis cases are required to be reported to FIDR by clinical microbiology laboratories [10]. The laboratories report all *Salmonella* cases diagnosed with culture, polymerase chain reaction (PCR), or other kinds of nucleic acid identification. A database was formed of salmonellosis notifications from 1 January 1995 to 31 December 2022. We characterized the cases based on age, gender, hospital district, nationality, travel history, and *Salmonella* serotype. Death within 30 days of sampling was considered *Salmonella*-related. We calculated a 5-year moving average of annual incidences for domestic, travel-related, and origin-unknown cases, as well as incidence rate ratios (IRRs) with 95% confidence intervals (CIs) for 5-year age groups, gender, and hospital districts as well as for incidences during the period 1995–1999 compared to 2015–2019. The years 2020–2022 were excluded from the comparison of time periods since case numbers were exceptionally low due to the COVID-19 pandemic.

We retrieved annual data on Finns’ travels abroad from 2012 to 2022 from Statistics Finland [11]. Travel data were missing for Africa in 2013–2018 and 2020–2022, for the Americas in 2020–2021, and for Asia and Oceania in 2021. The travel data for Africa for 2013–2018 were approximated based on the data from the years 2012 and 2019: there were 110,000 and 120,000 trips to Africa in 2012 and 2019, respectively. For the years with missing data, the number of trips was approximated to be the mean of the years 2012 and 2019 (115,000). We calculated incidences for cases who traveled to different regions of the world (cases/100,000 trips) and IRRs with 95% CI from 2012 to 2019. The years 2020–2022 were not included in the calculations, since both travel numbers and travel-related salmonellosis case numbers were exceptionally low due to the COVID-19 pandemic. Stata 18 software (StataCorp LLC, College Station, Texas, USA) was used to conduct statistical analyses.

### 2.2. Outbreaks

Municipal outbreak investigation groups in Finland report all suspected food- and waterborne outbreaks to a national food- and waterborne outbreak register (the FWO register) [12]. An outbreak reported to FWO register is defined by at least two cases having similar symptoms after exposure to a common source. We described reported foodborne outbreaks by causative agents and salmonellosis outbreaks according to number of cases, causative *Salmonella* serotype, and the source of the outbreak, from 1 January 2010 to 31 December 2022.

## 3. Results

### 3.1. Salmonella Surveillance

**Figure 1:**
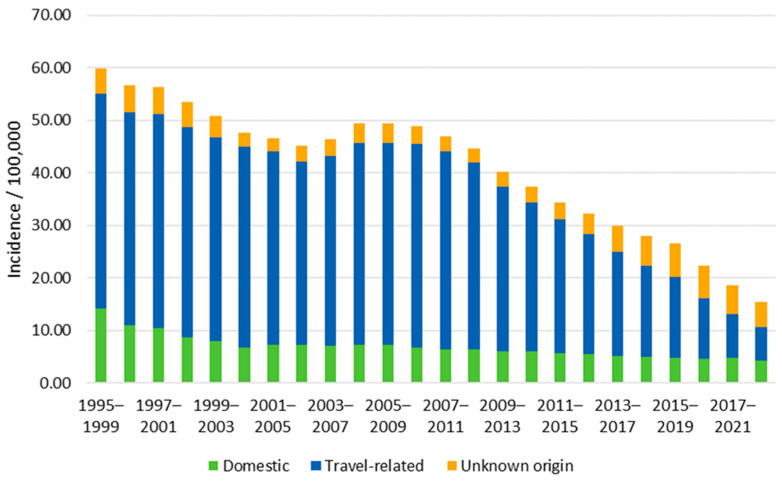
A five-year moving average of annual incidences for domestic, travel-related, and unknown origin salmonellosis cases in Finland from 1995–2022.

During 1995–2022, 60,002 salmonellosis cases were reported to the FIDR, of which 72% (43,062) were travel-related and 18% (10,975) of domestic origin. The travel history was unknown for 10% (5965) of the cases. Comparing the period 2015–2019 before the COVID-19 pandemic to the period 1995–1999, the incidence of salmonellosis decreased by 56% for all cases (IRR 0.44, 95% CI 0.43–0.45, *p*-value < 0.001) and by 66% and 63% for domestic (IRR 0.34, 95% CI 0.32–0.36, *p*-value < 0.001) and travel-related cases (IRR 0.37, 95% CI 0.36–0.39, *p*-value < 0.001), respectively (Figure 1). Of domestic and travel-related cases, 39 (0.4%) and 4 (0.01%), respectively, died within 30 days of sampling. The median ages of the deceased were 76 years (range of 38–94 years) for the domestic and 68 years (range of 59–77 years) for the travel-related cases, and 46% and 25% were female, respectively.

**Figure 2:**
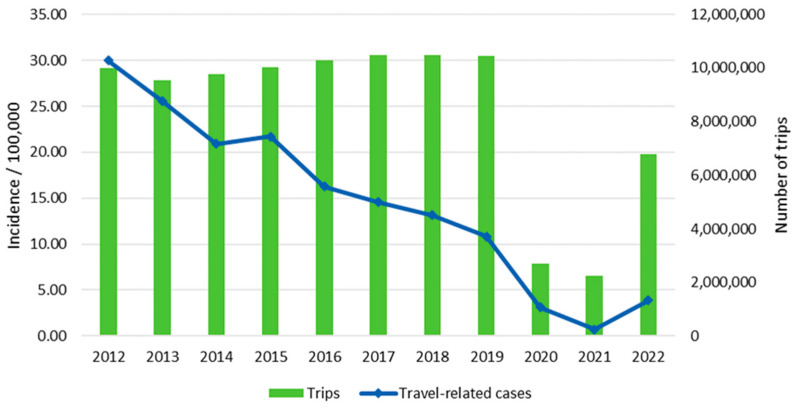
Incidence of travel-related salmonellosis cases and the number of trips abroad in Finland from 2012 to 2022.

In March 2020, the Finnish government declared a state of emergency due to the COVID-19 outbreak, and restrictions were made regarding traveling and the movement of humans. The number of travels abroad in the year 2020 decreased by 74% compared to the year 2019 (from 10,440,000 to 2,690,000; Figure 2). Subsequently, the incidence of travel-related salmonellosis in the year 2020 decreased by 71% compared to the year 2019 (from 10.86/100,000 to 3.11/100,000). The incidence of domestic and unknown origin salmonellosis in 2020 decreased by 2% and 62%, respectively, when compared to the year 2019.

The median ages of domestic and travel-related cases were 34 years (range < 1–100 years) and 38 years (range < 1–91 years), respectively. The incidence was two-fold among 0–4-year-old domestic cases and in 25–29-year-old travel-related cases (IRR 2.3, 95% CI 2.2–2.4, *p*-value < 0.001 and IRR 1.8, 95% CI 1.7–1.8, *p*-value < 0.001, respectively) compared to other age groups. There were slightly more females in both domestic (53%, 5861; IRR 1.1, 95% CI 1.1–1.1, *p*-value < 0.001) and travel-related cases (54%, 23,350; IRR 1.1, 95% CI 1.1–1.2, *p*-value < 0.001).

**Figure 3:**
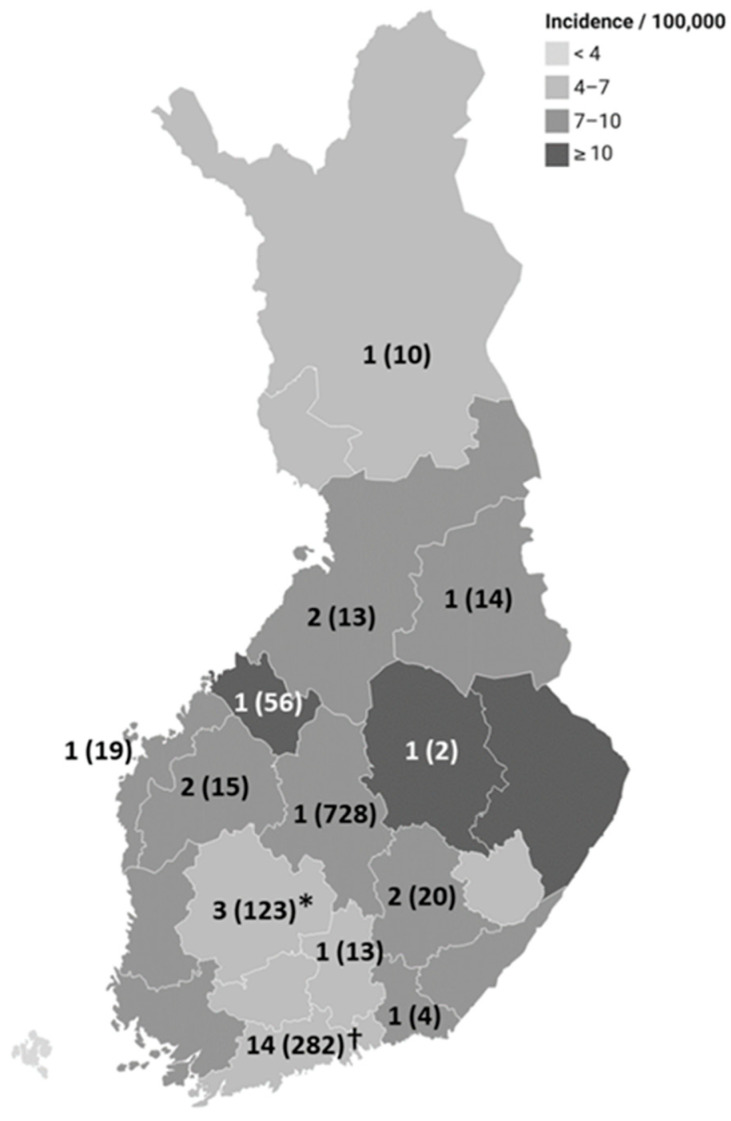
Incidence of domestic salmonellosis cases according to hospital district in Finland from 1995 to 2022. The number of foodborne *Salmonella* outbreaks reported to the Finnish food and waterborne outbreak register in 2010–2022 is indicated with numbers, and the number of cases is presented in brackets. Two outbreaks spanning several hospital districts are marked in the Helsinki and Uusimaa Hospital District (†), and one is in the Pirkanmaa Hospital District (*).

The highest incidences for domestic cases were observed in Central Ostrobothnia, North Karelia, and North Savo hospital districts compared to other hospital districts (IRR 1.5, 95% CI 1.4–1.7, *p*-value < 0.001; IRR 1.5, 95% CI 1.4–1.6, *p*-value < 0.001; IRR 1.4, 95% CI 1.3–1.5, *p*-value < 0.001, respectively) (Figure 3).

**Figure 4:**
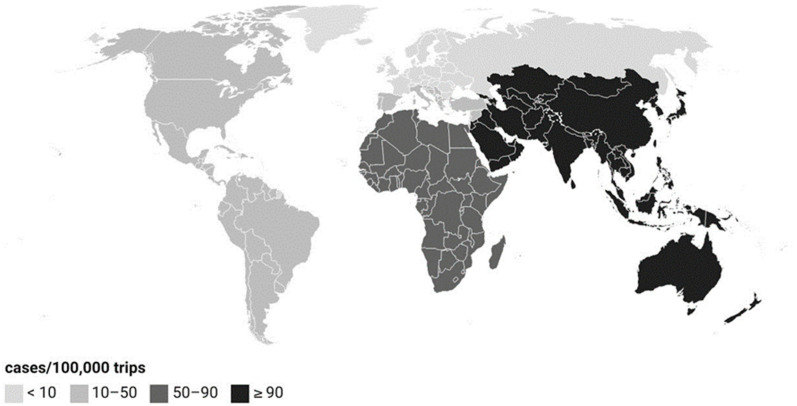
Incidence of Finland’s travel-related salmonellosis cases according to geographical region in 2012–2019. Regions: Nordic countries, Russia and Baltic countries, Eastern and Western Europe, Southern Europe and Eastern Mediterranean, the Americas, Africa, Asia, and Oceania.

Of the 43,062 travel-related cases, the most infections were contracted in Southeast Asia (27%, 11,775) and Southern Europe (19%, 8140). The most travel-related infections were contracted in Thailand (23%, 9774), Spain (10%, 4475), and Turkey (8%, 3603). From 2012 to 2019, the incidence rate was 22-fold for those who traveled to Asia and Oceania, and 9-fold for those who traveled to Africa when compared to people who traveled to other regions (IRR 22, 95% CI 22–23, *p*-value < 0.001, and IRR 9.9, 95% CI 8.5–9.8, *p*-value < 0.001, respectively; Figure 4).

**Figure 5:**
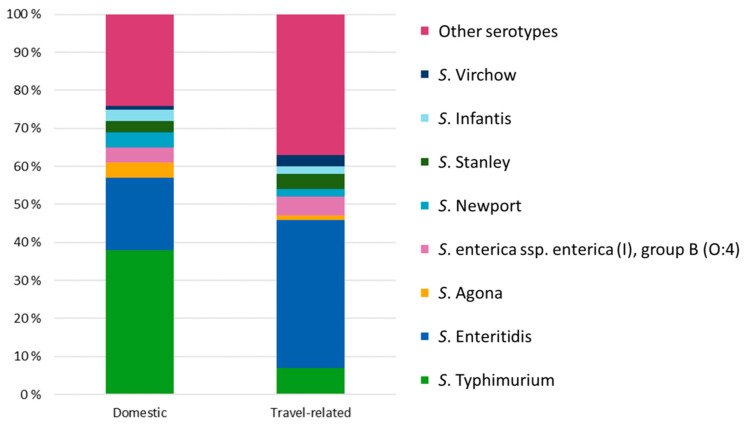
The proportion of serotypes in domestic (N = 10,975) and travel-related (N = 43,062) salmonellosis cases in Finland from 1995 to 2022.

In the period 1995–2022, 345 different *Salmonella* serotypes were reported. *S*. Enteritidis and *S*. Typhimurium were the most common serotypes (Figure 5). Of domestic cases, 38% were caused by *S*. Typhimurium and 19% by *S*. Enteritidis. Of travel-related cases, 39% were caused by *S*. Enteritidis and 7% by *S*. Typhimurium.

### 3.2. Foodborne Outbreaks

**Figure 6:**
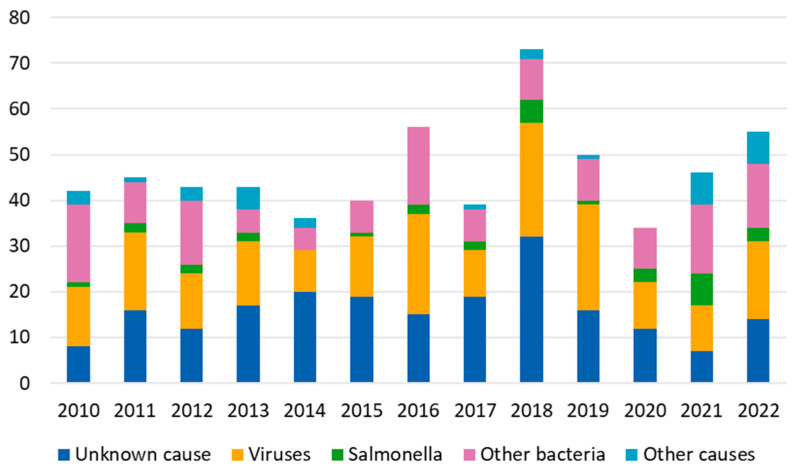
Number of annual foodborne outbreaks caused by *Salmonella* sp. and other causative agents reported to the national food- and waterborne outbreak register in Finland from 2010–2022.

In the period 2010–2022, 34–73 foodborne outbreaks were reported to the FWO register annually (Figure 6). Most outbreaks were caused by viruses (32%, 195/602), mainly norovirus, followed by bacteria (28%, 168/602). In 36% (219/602) of foodborne outbreaks, the causative agent remained unknown.

*Salmonella* sp. was reported as a cause in 31 foodborne outbreaks during the period 2010–2022 (Figure 6, Table 1). In these outbreaks, 1289 fell ill. In 14 outbreaks, the source was specified at food stuff level. In 14 outbreaks, the source was reported at the dining level, and, in 3 outbreaks, the source was unknown. Of the 14 outbreaks with a specified source, 11 (79%) were mediated by fresh or frozen vegetables, spices, or seeds of foreign origin (mung bean sprouts (2), zucchini (2), salad (1), ready-to-eat melon cubes (1), frozen tomato cubes (1), a salad containing foreign chopped iceberg lettuce and domestic fresh cucumber (1), arugula (1), spices (1), chia seeds (1)), 1 (7%) by beef stew, 1 (7%) by ready-to-eat products containing chicken of foreign origin, and 1 (7%) by raw milk of domestic origin. No waterborne outbreaks caused by *Salmonella* sp. were reported from 2010 to 2022.

## 4. Discussion

The reporting of salmonellosis has decreased during the 28-year surveillance period in Finland. Incidences of both travel-related and domestic salmonellosis have markedly declined, especially in the past 10 years. Salmonellosis in Finland is mainly travel-related, and the region of infection is most commonly Southeast Asia. Although the incidence of domestic salmonellosis has decreased, more foodborne outbreaks caused by *Salmonella* have been reported in recent years compared to the early 2010s.

In Finland, more than two-thirds of salmonellosis cases are of foreign origin, and a similar trend has been seen in other Nordic countries [13]. Traveler’s diarrhea originating from Southeast Asia and Africa has been found to be more common compared to other regions [14]. Although traveler’s diarrhea is a common problem among travelers in low-income countries, the rates have declined in recent decades [15]. The decline may be explained by improved hygienic standards and socioeconomic conditions in the destination countries [16]. The measures to prevent traveler’s diarrhea are the same as the ones for preventing salmonellosis in general [17]: Hands should be washed often with soap and water, especially before eating, after using the toilet, or having contact with animals or their feces. Foods should be thoroughly cooked and served hot, and foods in a cold buffet should be avoided. Raw fruits and vegetables should be peeled or washed with clean water. Beverages should be consumed from factory-sealed containers only, and ice cubes should be used only if made with clean water.

We observed a dramatic reduction in reported cases, mostly in travel-related salmonellosis, during the COVID-19-pandemic in 2020 and 2021. Similarly, the incidence of travel-related salmonellosis in Sweden decreased nearly fourfold from 2000 to 2019 [18] and pandemic-related reduction has also been reported in other countries such as the Netherlands and the UK [19,20]. For travel-related cases, this can be explained by restrictions on non-essential traveling abroad, and, for domestic cases, by restrictions on public gatherings [19,20].

In the EU/EEA area, the most common serovars infecting humans have been *S*. Enteritidis, followed by *S*. Typhimurium [21]. Similarly, *S*. Enteritidis and *S*. Typhimurium, in this order, were the most common serovars in travel-related cases in Finland. However, in domestic infections, *S*. Typhimurium was more common than *S*. Enteritidis. The order of serovars for domestic and travel-related cases in Finland is similar to that of Sweden and Norway [18,22]. In 2022 in the EU/EEA area, *S*. Enteritidis was most commonly associated with laying hen flocks and eggs and, secondly, with broiler flocks and meat [1]. In Finland, the incidence of salmonellosis in poultry is low, less than 1% [9], which could explain the smaller amount of *S*. Enteritidis in Finland’s domestic cases.

According to the national salmonella control program, routine *Salmonella* sampling in cattle and pigs focuses on slaughterhouses and cutting plants [5]. The aim is to keep the prevalence of salmonella in slaughtered cattle, sows, and fattening pigs at less than 1% [9], and in meat at less than 0.5% [5], which has been reached. During the period 2000–2023, *Salmonella* was found in under 0.3% of bovine and under 0.4% of pig lymph node samples tested, while the percentage of *Salmonella* positive carcass swab samples was below 0.3% in bovines and below 0.1% in pigs [23,24,25,26]. Since 2016 and 2007, no *Salmonella* has been found in domestic bovine and pig meat samples taken at meat cutting plants [23,24,25,26].

Although salmonellosis is rare in production animals in Finland, it occurs commonly among wild animals, such as hedgehogs and wild birds [27]. The most common *Salmonella* serotypes in hedgehogs are *S*. Enteritidis and *S*. Typhimurium. Similar *S*. Typhimurium strains have been found from humans and hedgehogs in Finland [28]. Wild hedgehogs have also been linked to human *S*. Typhimurium outbreaks in Norway [29]. In Finland, *S*. Typhimurium is often found from wild birds, especially gulls, and, in late winter, it causes deaths of songbirds at birdfeeders [27]. Epidemic deaths of songbirds caused by *S*. Typhimurium have been linked to human outbreaks in, for example, the United States [30] and New Zealand [31]. In Sweden, an outbreak of *S*. Typhimurium in cats linked to wild birds has been described with the indication of indirect transmission from birds to humans via sick cats [32]. Humans may contract salmonellosis from wild animals through handling ill or dead animals, coming into contact with surfaces contaminated with animal feces, or ingesting food or water contaminated with animal feces [33]. To reduce the risk of infection from wild animals, the feeding bowls and bird feeders should be kept clean; gloves should be worn when handling dead or live wild animals or their feces; and hands should be washed thoroughly with soap and water after any contact with the animals or the feeders [30]. Also, pets should be prevented from having contact with wild animals or their feces to reduce the risk of them getting infected with *Salmonella* and transmitting it to humans.

In our study, the differences in incidences between genders and hospital districts were small. Similarly to the EU/EEA area [21], the highest incidence in domestic cases was observed in children less than five years old. However, in the EU/EEA area, the incidence in under-5-year-olds was 10 times higher than that of adults, whereas, in Finland, it was only twice as high. The higher incidence in young children may be due to the fact that children have more frequently symptomatic infections, guardians are more prone to take young children to see a doctor, and doctors are more likely to take samples from sick children than from adults [21]. The case fatality rate in Finland was in line with that reported in EU/EEA area [1].

Even though the incidence of salmonellosis has decreased in Finland, more foodborne outbreaks caused by *Salmonella* have been reported in recent years compared to the early 2010s. Especially vegetables of foreign origin have mediated several outbreaks in Finland in recent years. In part, this may be due to the increased consumption of vegetables, fruit, and berries in Finland [34]. An increase in outbreaks mediated by fresh produce has also been observed in the U.S. [35]. The contamination of fresh produce is possible at all steps from farm to fork: cultivation, handling, processing, or preparation [35]. Several factors have been identified that affect the contamination of produce with *Salmonella*, for example, the use of contaminated irrigation water, agrotechnical mistakes made during cultivation, the lack of proper sanitation or hygienic practices during field work, and extreme weather events caused by climate change [36]. Good agricultural practices, recommendations, and standards have been developed by many organizations, but their communication to all actors in the supply chain, including the farm workers, could be strengthened [37]. After contamination, the intervention measures are more limited and more difficult to implement. Washing, with or without sanitizer, is an important part of produce preparation, especially of ready-to-eat produce [38]. However, it has been shown that washing does not necessarily reduce the bacterial load of leafy vegetables [39]. The greatest benefit would probably be in directing resources to prevent contamination, and to train and educate the growers, handlers, and consumers of handling fresh produce.

In the EU/EEA area in 2022, the most common food items implicated in strong-evidence salmonellosis foodborne outbreaks contained, for example, eggs and egg products, pig meat and meat products, and mixed foods [1]. The national salmonella control program that has been in place since 1995 has probably affected the low burden of *Salmonella* in food production animals in Finland. This can be seen in the low number of foodborne outbreaks mediated by domestic meat or eggs, indicating that the national salmonella control program is effective and important in preventing domestic human infections in Finland.

## Figures and Tables

**Table 1:** Foodborne *Salmonella* outbreaks reported to the food and waterborne outbreak register in Finland in the period 2010–2022.

Year	Serotype	People Fallen Ill	Source
2010	*S*. Typhimurium	10	Salad
2011	*S*. Typhimurium	2	Unknown
2011	*S*. Oranienburg	15	Unknown
2012	*S*. Agona	97	Dining at summer party
2012	*S*. Infantis	13	Dining at a restaurant
2013	*S*. Typhimurium	9	Raw milk
2013	*Salmonella* sp.	4	Dining at a restaurant
2015	*S*. Newport	45	Chia seed
2016	*Salmonella* sp.	3	Dining at home
2016	*S*. Enteritidis	22	Mung bean sprouts
2017	*S*. Enteritidis	32	Mung bean sprouts
2017	*S.* Bareilly	23	Spices
2018	*S*. Newport	15	Dining at hospital
2018	*S*. Agama	14	Beef stew
2018	*Salmonella* sp.	4	Dining at a restaurant
2018	*S*. Newport	19	Dining at a café
2018	*S*. Newport	4	Unknown
2019	*S*. Poona	9	Ready-to-eat melon cubes
2020	*S*. Agona	4	Dining at residential care
2020	*S*. Kedougou	7	Zucchini
2020	*S*. Saintpaul	10	Dining at school/kindergarten
2021	*S*. Bareilly	4	Dining at a restaurant
2021	*S*. Enteritidis	12	Dining at a restaurant
2021	*S*. Enteritidis	2	Dining at a restaurant
2021	*S*. Enteritidis	4	Dining at a restaurant
2021	*S*. Kedougou	13	Zucchini
2021	*S*. Typhimurium	56	Frozen tomato cubes
2021	*S*. Typhimurium	728	Fresh cucumber/chopped iceberg lettuce
2022	*S*. Mbandaka	97	Ready-to-eat products containing chicken
2022	*S*. Typhimurium	6	Arugula
2022	*Salmonella* sp.	6	Dining at a restaurant

## Data Availability

The raw/processed data analyzed in this study cannot be shared due to the European General Data Protection Regulation.
